# Cancer Cell-Derived Small Extracellular Vesicles Are Associated with Impaired C2C12 Myoblast Differentiation

**DOI:** 10.3390/ijms27177894

**Published:** 2026-09-04

**Authors:** Woojin Lee, Seongjae Yang, Hyeonseo Yu, Yeongmin Kim, Seongmin Cho, Hyeong Yun Kim, Uijoo Kim, Hyunseok Kong, Sang Bum Kim

**Affiliations:** 1 College of Pharmacy, Sahmyook University, Seoul 01795, Republic of Korea; 2 Institute for Artificial Intelligence and Biomedical Research (AIBI), Medicinal Bioconvergence Research Center, College of Pharmacy, Yonsei University, Incheon 21983, Republic of Korea; 3 KOSA BIO Inc., 272, Namyangju-si 12106, Republic of Korea

**Keywords:** cancer cachexia, small extracellular vesicles, myoblast differentiation, myogenic regulatory factors, myosin heavy chain

## Abstract

Cancer cachexia is a complex metabolic syndrome characterized by progressive skeletal muscle wasting and poor clinical outcomes. Previous studies have mainly focused on the association between cancer-derived extracellular vesicles and atrophy of differentiated myotubes, whereas their effects on myoblast differentiation markers have not been sufficiently elucidated. In this exploratory in vitro study, vesicle fractions isolated from MC38, CT26, MCA205, and B16F10 cancer cell lines under serum-deprived conditions were referred to as small extracellular vesicles (sEVs) and used to treat C2C12 myoblasts during differentiation. Compared with the control group, the sEV-treated groups showed reduced myotube diameter and differentiation area on day 6, while the mRNA expression patterns of *MyoD*, *MyoG* (*Myogenin*), *MRF4*, *Myf5*, *Pax7*, and several MHC isoforms showed time-dependent changes. GO/KEGG analysis of commonly identified proteins revealed enrichment of terms related to mRNA metabolism, the cell cycle, and the proteasome. These findings suggest that treatment with cancer cell-derived sEVs may be associated with impaired in vitro differentiation of C2C12 myoblasts.

## 1. Introduction

Cancer cachexia is characterized by progressive skeletal muscle wasting in patients with cancer and cannot be completely reversed by conventional nutritional support [1,2]. Patients with cancer cachexia often exhibit reduced tolerance to chemotherapy and diminished responsiveness to anticancer treatment. Cachexia occurs in a substantial proportion of patients with cancer and contributes directly to cancer-related mortality [3,4,5]. Major mechanisms driving muscle wasting in cancer cachexia involve tumor-derived cytokines such as interleukin-6 (IL-6), tumor necrosis factor-α (TNFα), and interferon-γ (IFNγ), which disrupt protein metabolism, promote catabolism, increase energy expenditure, and mobilize nutrients into the circulation, ultimately leading to skeletal muscle breakdown [1,6,7,8]. Recent studies have also shown that cancer cell-derived small extracellular vesicles (sEVs) can directly affect skeletal muscle. Treatment of differentiated C2C12 myotubes with cancer-derived sEVs induces protein degradation and apoptosis and reduces myotube diameter, suggesting that sEVs may contribute to muscle atrophy in cancer cachexia [2,9,10,11,12,13].

Extracellular vesicles are membrane-bound particles released by most cell types and contain proteins, lipids, and nucleic acids [14]. Their size distribution and biological activity vary according to cellular origin and culture conditions [14,15,16,17,18]. Because the isolates in this study had mean NTA sizes of 148.9–154.5 nm and their endosomal origin was not directly demonstrated, they are hereafter described as sEVs. Cancer-derived sEVs may be associated with tumor progression and skeletal muscle homeostasis [19].

Adult skeletal muscle has a robust regenerative capacity that relies on muscle satellite cells activated in response to injury or disease [19,20]. Activated satellite cells express Pax7 and generate myoblasts [21], which subsequently undergo differentiation and fusion under the control of myogenic regulatory factors (MRFs), including Myf5, MyoD, MRF4, and myogenin (MyoG) [22,23,24]. Myf5 and MyoD are important for myoblast lineage determination and early differentiation, whereas MRF4 and MyoG promote myotube formation. During late differentiation, myosin heavy chain (MHC) and myosin light chain proteins accumulate to support final myofiber maturation [25]. Previous studies have mainly focused on the detrimental effects of cancer-derived sEVs on differentiated myotubes, whereas their effects on myoblasts, which are essential for skeletal muscle repair and maintenance, remain largely unknown.

In this study, we conducted a descriptive, exploratory in vitro study to investigate whether treatment with cancer cell-derived sEVs isolated under serum-deprived conditions [26,27,28], which may reflect adaptive stress phenotypes associated with advanced malignancy, was associated with changes in in vitro differentiation markers in C2C12 myoblasts. Using C2C12 cells, an established in vitro model of myogenic differentiation [29,30], we evaluated myotube formation, MRF expression, and MHC isoform expression over time. We also performed mass spectrometry and GO/KEGG analyses of proteins shared among the sEV isolates to generate hypotheses for subsequent validation.

## 2. Results

### 2.1. Characterization of Cancer Cell-Derived sEVs

**Figure 1:**
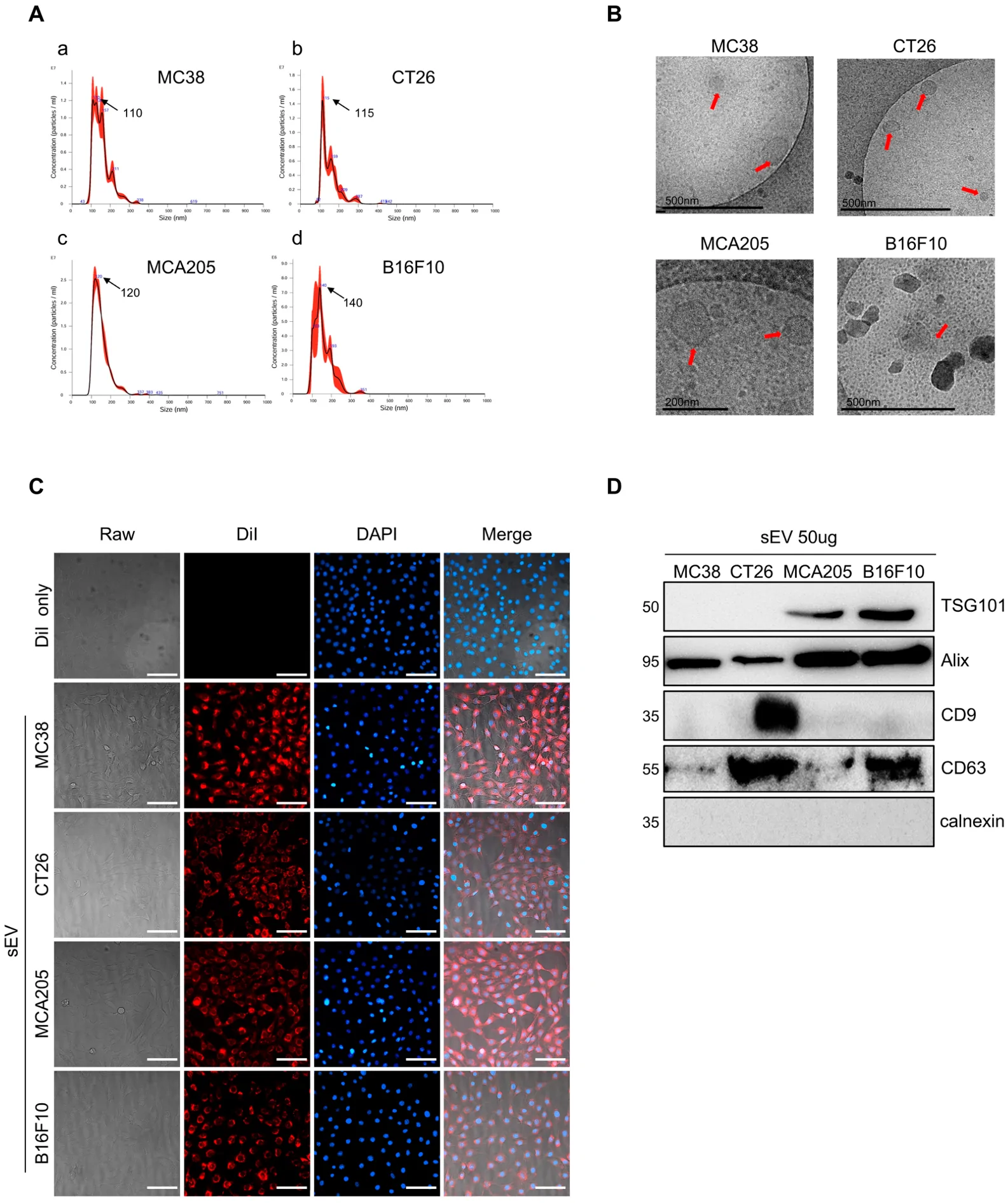
Characterization of cancer cell-derived sEVs. (**A**) Nanoparticle tracking analysis (NTA) of cancer cell-derived sEVs. The mean particle sizes were 153.7 ± 2.1 nm, 151.7 ± 3.1 nm, 148.9 ± 0.6 nm, and 154.5 ± 1.4 nm for MC38- (**a**), CT26- (**b**), MCA205- (**c**), and B16F10-derived sEVs (**d**), respectively. D10, D50, and D90 represent the particle diameters corresponding to 10%, 50%, and 90% of the cumulative distribution, respectively. The black line indicates the particle size corresponding to the highest peak of the distribution. (**B**) Representative cryo-electron microscopy (cryo-EM) images of cancer cell-derived sEVs. Scale bars, 500 nm for MC38, CT26, and B16F10-derived sEVs and 200 nm for MCA205-derived sEVs. Red arrows indicate the sEVs in the cryo-EM images. (**C**) C2C12 myoblasts treated with DiI-labeled sEVs, with nuclei counterstained with DAPI, showing the uptake of cancer cell-derived sEVs. Scale bar, 100 μm. (**D**) Western blot analysis of sEV-associated markers, including TSG101, Alix, CD63, CD9, and the cellular protein marker Calnexin.

To investigate the effects of sEVs on myoblasts, sEVs were isolated from mouse cancer cell lines, including colon cancer cells (MC38 and CT26), fibrosarcoma cells (MCA205), and melanoma cells (B16F10), and subsequently applied to mouse C2C12 myoblasts. The isolated sEVs were characterized based on their size distribution, morphology, and expression of extracellular vesicle-associated markers. Calnexin was included as a cellular protein marker to assess potential contamination with cellular components. Uptake of sEVs by myoblasts was further evaluated by labeling the sEVs with DiI dye and observing recipient cells using fluorescence microscopy [31]. Nanoparticle tracking analysis (NTA) showed mean particle sizes of 153.7 nm for MC38 (D10/D50/D90: 105.7/143.5/215.1 nm), 151.7 nm for CT26 (107.3/137.5/212.3 nm), 148.9 nm for MCA205 (105.8/137.3/201.8 nm), and 154.5 nm for B16F10 (103.9/143.8/214.7 nm) (Figure 1A). Cryo-electron microscopy (cryo-EM) confirmed the characteristic lipid bilayer morphology of the isolated sEVs (Figure 1B). To assess sEV internalization, C2C12 myoblasts were treated with DiI-labeled sEVs following DAPI staining of the nuclei. Fluorescence microscopy revealed DiI-positive signals surrounding the nuclei, indicating the uptake of cancer cell-derived sEVs by C2C12 myoblasts (Figure 1C). Western blot analysis demonstrated that Alix was consistently detected in all sEV preparations, whereas the expression levels of CD63, TSG101, and CD9 varied among sEVs derived from different cancer cell lines. Equal amounts of total protein (50 μg per sample) were loaded for Western blot analysis, and protein loading was assessed by Ponceau S staining in Supplementary Figure S1. CD63 signals were relatively weaker in MC38- and MCA205-derived sEVs, whereas CD9 was predominantly detected in CT26-derived sEVs. TSG101 was detected only in MCA205- and B16F10-derived sEVs, while Alix expression was relatively lower in MC38- and CT26-derived sEVs. Calnexin was not detected in any of the preparations, suggesting minimal contamination with cellular components (Figure 1D).

### 2.2. Cancer Cell-Derived sEVs Inhibit C2C12 Myoblast Differentiation

**Figure 2:**
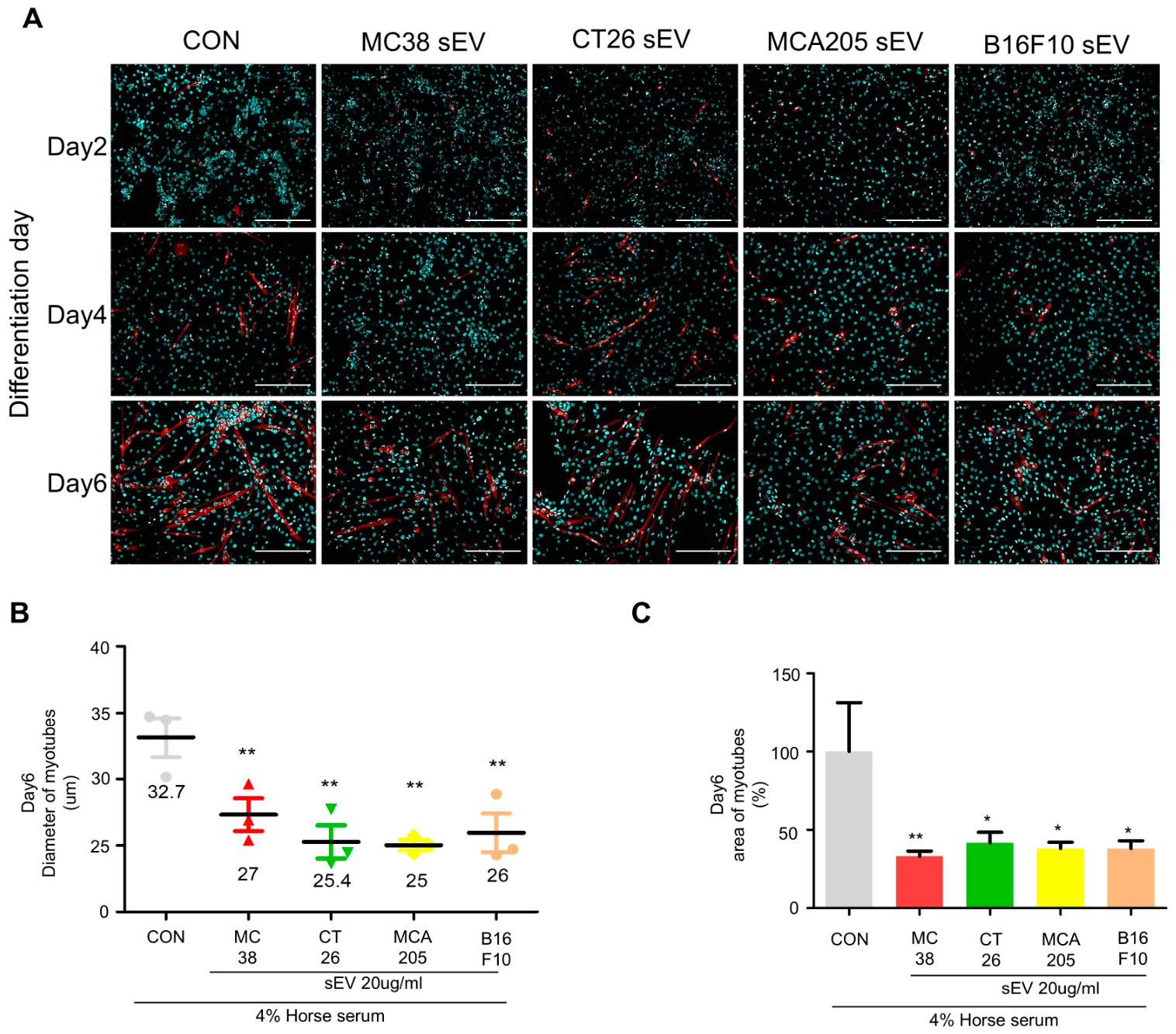
Cancer cell-derived sEVs inhibit C2C12 myoblast differentiation. (**A**) Immunofluorescence staining was performed to evaluate C2C12 myoblast differentiation on days 2, 4, and 6 following treatment with cancer cell-derived sEVs. Scale bar, 200 μm. (**B**,**C**) Myotube diameter and MHC-positive myotube area percentage were quantified. Data are presented as mean ± SEM from three independent biological replicates (*n* = 3). Statistical comparisons were performed between each sEV-treated group and the corresponding untreated control at the same differentiation time point. * *p* < 0.05 and ** *p* < 0.01.

Myoblast differentiation into multinucleated myotubes is regulated by myogenic regulatory factors (MRFs) and is accompanied by increased MHC expression during myotube maturation. To determine whether cancer cell-derived sEVs affect C2C12 myoblast differentiation, immunofluorescence staining was performed using an anti-MHC antibody to evaluate myotube formation, diameter, and differentiation area [32]. C2C12 myoblasts were induced to differentiate under horse serum-containing conditions for up to 6 days [33]. Reduced MHC-positive myotube formation became apparent from day 4 in the sEV-treated groups. By day 6, myotube formation was markedly reduced in all cancer cell-derived sEV-treated groups. Representative immunofluorescence images showed reduced myotube formation in B16F10- and MCA205-derived sEV-treated groups, whereas relatively thin myotubes were observed in MC38- and CT26-derived sEV-treated groups compared with the control group (Figure 2A). Quantitative analysis further showed significant reductions in myotube diameter and the percentage of MHC-positive myotube area in all treated groups (Figure 2B,C). Three independent biological replicates were analyzed, with measurements within each replicate averaged to obtain a single value for statistical analysis. To determine whether this inhibitory effect was associated with the cancer cell origin of the sEVs, sEVs derived from non-malignant cells were evaluated under the same differentiation conditions. In contrast to cancer cell-derived sEVs, non-malignant cell-derived sEVs did not produce a comparable inhibitory effect on C2C12 myoblast differentiation (Supplementary Figure S2). In addition, Annexin V and SYTOX Green assays were performed to assess cellular stress and cell death under the serum-free conditions used for sEV preparation, with the results presented in Supplementary Figure S3. Collectively, these findings indicate that cancer cell-derived sEVs are associated with impaired C2C12 myoblast differentiation, as evidenced by reduced MHC-positive myotube formation, myotube diameter, and differentiation area. The lack of a comparable inhibitory effect of non-malignant cell-derived sEVs further supports an association between the observed phenotype and the cancer cell origin of the sEVs (Supplementary Figure S2).

### 2.3. Cancer Cell-Derived sEVs Alter Myogenic Regulatory Factor Expression

**Figure 3:**
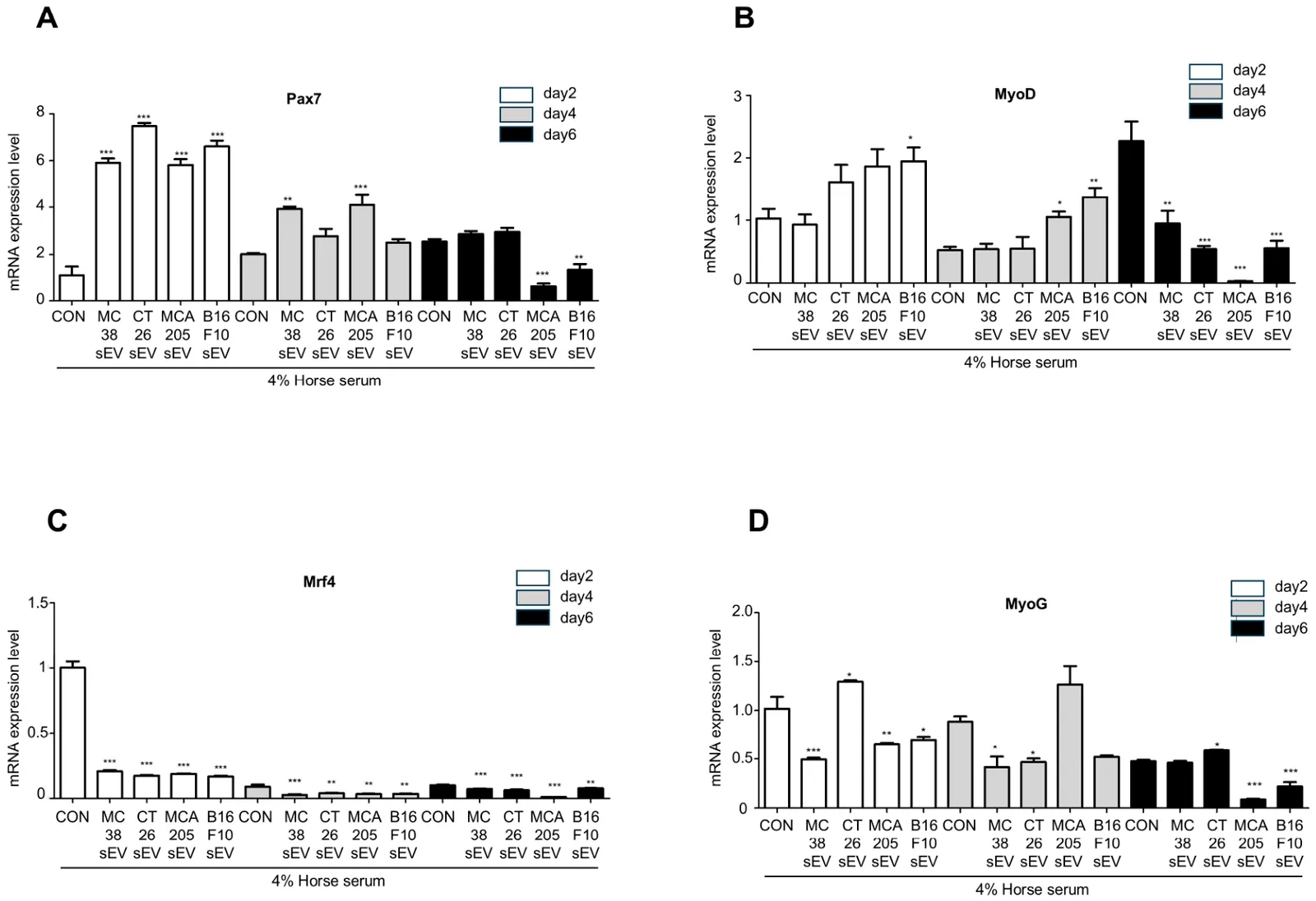
Effects of cancer cell-derived sEVs on myogenic regulatory factor expression. qRT-PCR analysis was performed using the 2^−ΔΔCt^ method. Expression levels of (**A**) *Pax7*, (**B**) *MyoD*, (**C**) *MRF4*, and (**D**) *MyoG*, were assessed at the indicated differentiation time points. Data are presented as mean ± SEM from three independent biological replicates (*n* = 3). Statistical comparisons were performed between each sEV-treated group and the corresponding untreated control at the same differentiation time point. * *p* < 0.05, ** *p* < 0.01, and *** *p* < 0.001.

Skeletal muscle differentiation is regulated by a coordinated network of myogenic regulatory factors (MRFs), including MyoD, Myf5, MyoG, and MRF4 [34,35]. These transcription factors are expressed in a temporally regulated manner and contribute to myoblast commitment, differentiation, and fusion. Pax7 is associated with myogenic precursor cell activation and is involved in myoblast proliferation and differentiation [21]. Following myogenic activation, MyoD and Myf5 contribute to myogenic commitment, whereas MyoG and MRF4 are more closely associated with later stages of differentiation and myotube formation [24,36]. To characterize changes in myogenic regulatory gene expression during C2C12 differentiation, the expression levels of *Pax7*, *MyoD*, *MRF4*, and *MyoG* were examined by qRT-PCR at days 2, 4, and 6 following differentiation induction. qRT-PCR and immunofluorescence analyses were performed using separate parallel cultures treated with the same sEV preparations. Thus, the gene expression changes were interpreted as being associated with the observed differentiation phenotype rather than as direct explanations of the morphological changes. *Pax7* expression was significantly increased in all sEV-treated groups on day 2 compared with the control group. Thereafter, this significant increase persisted only in the MC38 and MCA205 groups on day 4, and in the MCA205 and B16F10 groups on day 6 (Figure 3A). *MyoD* expression was increased in the MCA205- and B16F10-sEV-treated groups on day 4 but was decreased in sEV-treated groups on day 6 (Figure 3B). *MRF4* expression was decreased in sEV-treated groups on day 6, particularly in the MCA205-sEV-treated group (Figure 3C). *MyoG* expression was decreased in the MC38-, CT26-, and B16F10-sEV-treated groups on day 4 and in the B16F10- and MCA205-sEV-treated groups on day 6 (Figure 3D). Overall, cancer cell-derived sEVs induced time- and treatment-dependent alterations in myogenic regulatory gene expression during C2C12 differentiation. Although the magnitude and timing of these changes varied among sEV treatments, decreases in *MyoD*, *MRF4*, and *MyoG* were observed in several treatment groups, particularly at later stages of differentiation. These molecular changes were consistent with the impaired myogenic differentiation phenotype observed by immunofluorescence but do not establish a direct causal relationship between individual MRF changes and the observed morphological phenotype.

### 2.4. Cancer Cell-Derived sEVs Suppress MHC Isoform Expression

MHC is expressed during the later stages of muscle differentiation and contributes to muscle contractile properties and function. MHC isoforms are broadly classified into type I and type II isoforms, which exhibit distinct expression patterns during muscle fiber maturation [37]. Because immunofluorescence analysis showed reduced MHC expression and impaired myotube formation (Figure 2A), we further examined the mRNA expression of MHC isoforms by qRT-PCR using separate parallel cultures treated with the same sEV preparations.

**Figure 4:**
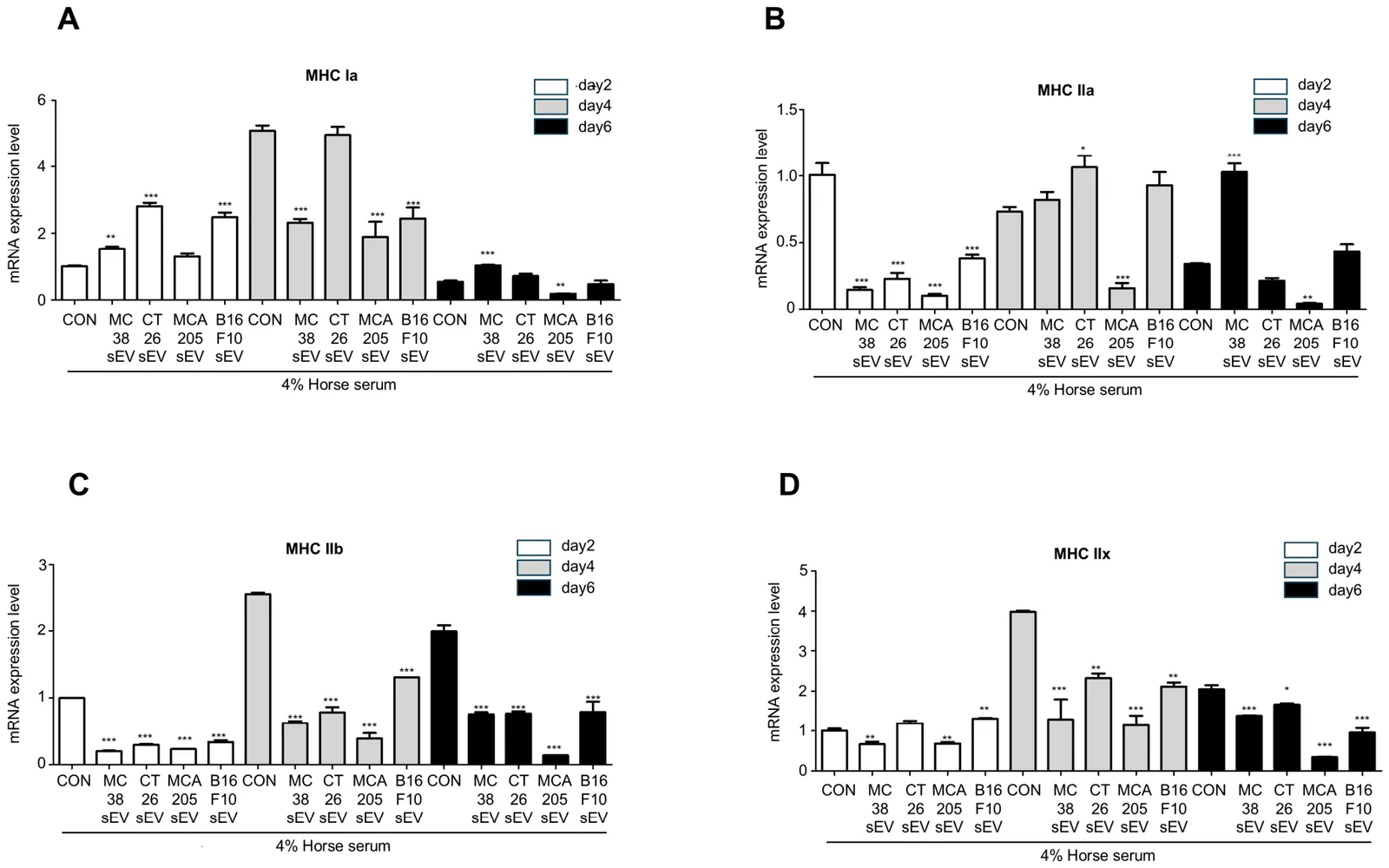
Effects of cancer cell-derived sEVs on *MHC* isoform mRNA expression during differentiation. qRT-PCR analysis was performed using the 2^−ΔΔCt^ method on days 2, 4, and 6. Expression levels of (**A**) *MHC Iα*, (**B**) *MHC IIa*, (**C**) *MHC IIb*, and (**D**) *MHC IIx* were assessed. Data are presented as mean ± SEM from three independent biological replicates (*n* = 3). Statistical comparisons were performed between each sEV-treated group and the corresponding untreated control at the same differentiation time point. * *p* < 0.05, ** *p* < 0.01, and *** *p* < 0.001.

*MHC Iα* expression was reduced on day 4 in all sEV-treated groups except the CT26 sEV-treated group compared with the corresponding untreated control (Figure 4A). *MHC IIa* expression was also decreased compared with the corresponding control on day 2 (Figure 4B). In addition, *MHC IIb* and *MHC IIx* expression were significantly decreased in all sEV-treated groups on days 4 and 6 (Figure 4C,D). Among these isoforms, *MHC IIb* showed a marked reduction across the sEV-treated groups, consistent with its relatively high abundance in mouse skeletal muscle [38].

Overall, cancer cell-derived sEVs were associated with time- and treatment-dependent changes in *MHC* isoform expression during C2C12 differentiation, with reductions in several *MHC* isoforms being particularly evident at later differentiation stages. These molecular changes were associated with the reduced *MHC* expression and impaired myotube formation observed by immunofluorescence analysis and were consistent with impaired progression of muscle differentiation and maturation.

### 2.5. Proteomic Analysis of Cancer Cell-Derived sEVs

To characterize the protein composition of cancer cell-derived sEVs isolated under serum-free conditions, proteins were analyzed by RP-nano liquid chromatography–electrospray ionization tandem mass spectrometry. Based on protein accession IDs, 1677 proteins were identified in MC38 sEVs, 1608 in CT26 sEVs, 2584 in MCA205 sEVs, and 2156 in B16F10 sEVs. A total of 425 protein IDs were commonly detected across all four sEV preparations based on a Sequest HT score greater than 10 and a minimum of two unique peptides.

**Figure 5:**
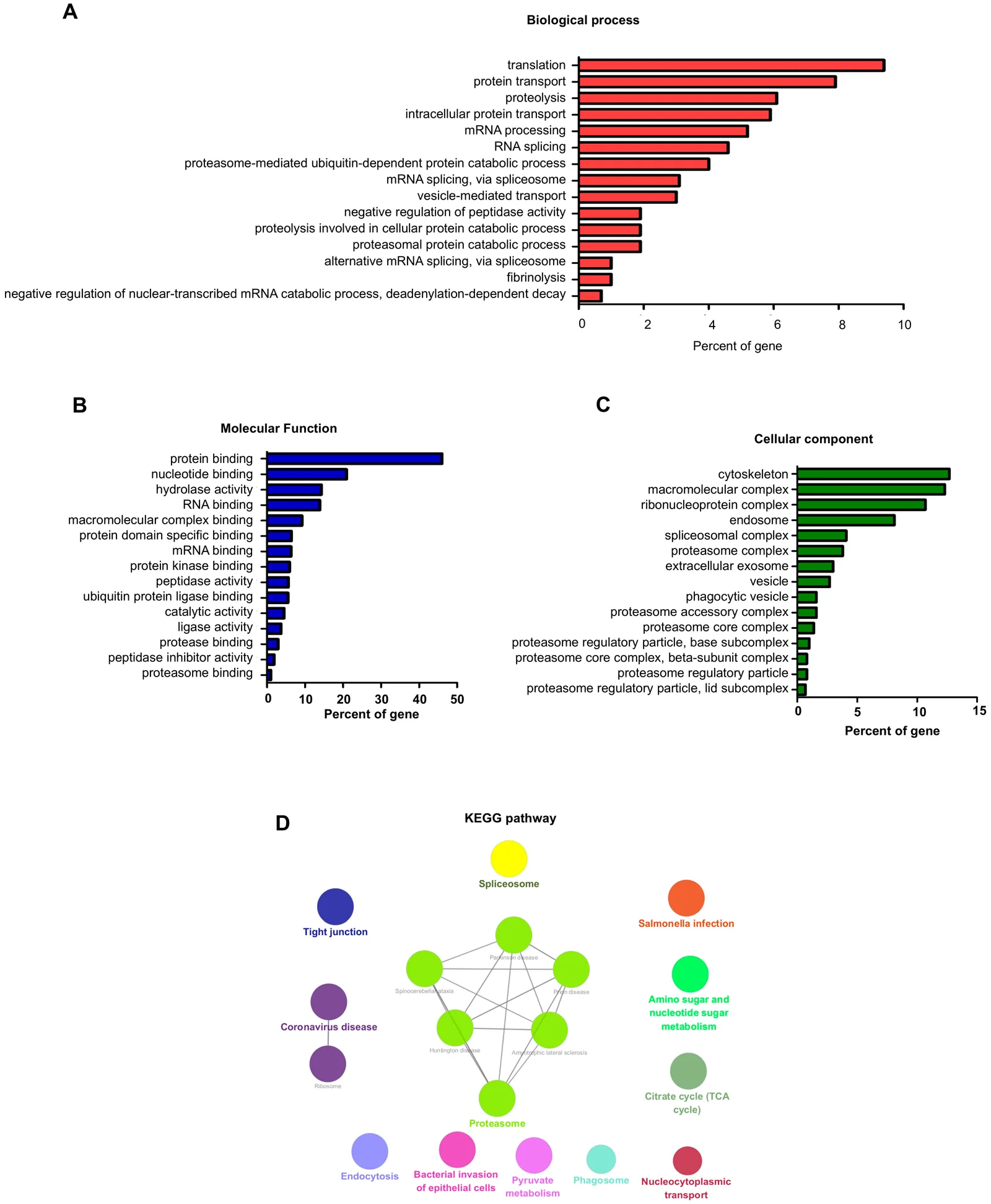
Gene Ontology and KEGG pathway analyses shared cancer cell-derived sEV proteins. Shared proteins identified in cancer cell-derived sEVs were analyzed using DAVID and categorized according to (**A**) biological process, (**B**) cellular component, (**C**) molecular function, and (**D**) KEGG pathways.

Corresponding gene IDs were analyzed using the Database for Annotation, Visualization, and Integrated Discovery (DAVID). Gene Ontology (GO) enrichment analysis was performed for molecular function, cellular component, and biological process terms using a significance threshold of *p* < 0.001. GO analysis identified multiple terms related to mRNA metabolism and cell-cycle regulation (Figure 5A–C). Kyoto Encyclopedia of Genes and Genomes (KEGG) pathway analysis further identified enrichment of proteasome-related pathways (Figure 5D). These findings indicate that proteins associated with several biological processes and pathways, including the proteasome, were commonly detected in cancer cell-derived sEVs (Table 1).

## 3. Discussion

Numerous molecular pathways have been implicated in cancer cachexia-associated muscle wasting; however, effective therapeutic approaches remain limited [39]. This limitation reflects the multifactorial nature of cachexia, which involves metabolic, inflammatory, and tissue-regenerative disturbances. Most previous studies have focused on the reduction of muscle size in differentiated myotubes. In contrast, few studies have examined whether cancer-derived factors impair myoblast self-renewal or differentiation processes that are essential for skeletal muscle maintenance and repair. The present study provides evidence that sEVs derived from cancer cell lines known to induce muscle wasting, including CT26, MCA205, and B16F10 [40,41], as well as MC38, are associated with impaired myogenic differentiation. Notably, MC38 has not been classically characterized as a cachexia-inducing tumor model, yet MC38-derived sEVs produced inhibitory effect on myogenic differentiation in our in vitro assay. This finding suggests that the ability of cancer cell-derived sEVs to interfere with myogenic differentiation may not be restricted to cancer models with established in vivo cachectic phenotypes. But these findings should not be interpreted as evidence that MC38-derived sEVs induce cancer cachexia in vivo, rather as evidence that cancer-derived sEVs can interfere with myogenic differentiation under the specific conditions tested.

In addition, sEVs were isolated under serum-free conditions. Solid tumors can survive and proliferate in nutrient-deprived environments during tumor progression, metastasis, and chemotherapy by activating adaptive survival mechanisms [26,27,28]. Such stress conditions can alter tumor cell phenotypes and promote aggressive cellular behavior. Serum depletion in cancer cell cultures has also been reported to increase autoregenerative or stem-like subpopulations [42]. Thus, cancer cell-derived sEVs produced under serum-depleted conditions may represent vesicles released from stress-adapted cancer cells and may have distinct biological effects on recipient myoblasts. However, serum-deprived culture conditions may also alter sEV composition and biological activity, and the contribution of the culture condition itself cannot therefore be excluded.

Patients with cancer cachexia experience skeletal muscle damage due to chemotherapy, inflammatory cytokines, and systemic metabolic stress [1]. Skeletal muscle normally regenerates efficiently through the activation of satellite cells [43]. *Pax7* is required for satellite cell activation and self-renewal; however, persistent *Pax7* expression can inhibit myogenic differentiation [44]. In agreement with this concept, sEV-treated cells showed increased *Pax7* expression at early time points. Activated Pax7-positive cells may proceed toward proliferation through MyoD and Myf5 activation, whereas Pax7-positive/MyoD-negative cells resemble quiescent or reserve satellite-like cells [45,46,47]. Subsequent differentiation requires MyoG induction, and *MRF4* is expressed during later stages of myoblast fusion and myotube maturation [34]. In the present study, cancer-derived sEVs decreased *MRF4* expression during differentiation. *MRF4* is expressed in differentiated muscle and during early muscle development [23] and has been proposed to function upstream of *MyoD* under specific developmental contexts [48]. Therefore, suppression of MRF4 and MyoD may contribute to inefficient myogenic differentiation, consistent with previous evidence that MyoD-deficient myogenic cells exhibit impaired differentiation capacity [49]. Nevertheless, because MRFs are interconnected in a context-dependent regulatory network, the causal hierarchy among Pax7, MyoD, MyoG, and MRF4 requires further mechanistic validation.

Skeletal muscle fibers are composed of sarcomeres, in which actin–myosin interactions generate contractile force. MHC is a major determinant of sarcomere function and muscle fiber characteristics [38,50]. Type I MHC is associated with slow-twitch oxidative fibers and fatigue resistance, whereas type II MHC isoforms are associated with fast-twitch fibers that develop predominantly during later myotube formation [51,52]. As myogenic differentiation progresses, MRF activation promotes MHC expression and myosin assembly [53]. In this study, cancer cell-derived sEVs generally decreased type II MHC expression, with notable reductions in *MHC Iα* and *MHC IIb*. This pattern may contribute to reduced myotube thickness and impaired contractile maturation. However, interpretation should consider species-specific variation and compensatory regulation among MHC isoforms [38]. Interestingly, CT26-derived sEVs did not reduce *MHC Iα* expression on day 4, whereas myotube diameter was reduced by day 6. This suggests that early differentiation may not be uniformly affected by all sEV sources, whereas suppression of type II MHC may contribute to later impairment of myotube maturation.

The proteomic analysis identified shared sEV-associated proteins enriched in proteasome-related pathways. However, these findings should be considered exploratory rather than mechanistic evidence. Although the ubiquitin-proteasome system regulates skeletal muscle homeostasis, stem cell maintenance, and atrophy-related protein turnover [42,54], the present proteomic analysis did not identify specific sEV-associated proteins that could be directly linked to the observed inhibition of myogenic differentiation, nor were candidate proteins functionally validated. Therefore, the proteomic enrichment results should be interpreted as hypothesis-generating findings rather than evidence of a direct mechanistic role of proteasome-associated sEV cargo in the observed impairment of myogenic differentiation. Further studies combining quantitative proteomic analysis with validation of individual candidate proteins and functional gain- or loss-of-function experiments will be required to determine whether specific sEV cargo directly contributes to altered myogenic transcriptional programs, stress-response pathways, or protein-homeostasis pathways.

## 4. Materials and Methods

### 4.1. Cell Culture

Murine C2C12 myoblasts were purchased from the American Type Culture Collection (ATCC). B16F10 melanoma cells, human embryonic kidney (HEK293) cells, mouse Leydig (TM3) cells, and human lung fibroblast (MRC-5) cells were obtained from the Korean Cell Line Bank (KCLB). MCA205 fibrosarcoma cells were purchased from Sigma-Aldrich. MC38 colon adenocarcinoma cells, CT26 colon carcinoma cells, and human keratinocyte (HaCaT) cells were kindly provided by Professor Sunghoon Kim (College of Pharmacy, Yonsei University, Incheon, Republic of Korea).

C2C12, MC38, B16F10, HEK293, and HaCaT cells were cultured in Dulbecco’s Modified Eagle’s Medium (DMEM), whereas MRC-5 cells were maintained in Eagle’s Minimum Essential Medium (EMEM), TM3 cells in Ham’s F-12 medium, and MCA205 and CT26 cells in Roswell Park Memorial Institute 1640 medium (RPMI-1640; all from HyClone, Logan, UT, USA). Unless otherwise specified, all culture media were supplemented with 10% heat-inactivated fetal bovine serum (FBS; HyClone) and 1% penicillin/streptomycin (P/S; Gibco, Waltham, MA, USA).

To induce C2C12 myoblast differentiation, cells were cultured until they reached 70–80% confluence. The culture medium was then replaced with differentiation medium (DM) consisting of DMEM supplemented with 4% heat-inactivated horse serum (Gibco) and 1% P/S. The medium was changed every two days, and differentiation was maintained for six days. All cell lines were incubated at 37 °C in a humidified 5% CO_2_ atmosphere (ICO 240 Eco; Memmert, Schwabach, Germany).

### 4.2. Small Extracellular Vesicle (sEV) Purification

Cells were cultured to approximately 80% confluence, after which the medium was replaced with serum-free DMEM containing 1% P/S and the cells were incubated for 24 h. Conditioned medium was collected and centrifuged at 1000× *g* for 10 min at 4 °C to remove dead cells and debris. The supernatant was then centrifuged at 10,000× *g* for 35 min at 4 °C to remove cell fragments and large vesicles. The resulting supernatant was concentrated using a 100 kDa Amicon filter (Merck, Darmstadt, Germany), followed by ultracentrifugation at 100,000× *g* for 70 min at 4 °C. The pellet was resuspended in DMEM, and protein concentration was measured using a BCA protein assay kit (Thermo Fisher Scientific, Waltham, MA, USA) before use. For each cell line, sEVs were independently isolated for each experimental series, aliquoted, and stored at −80 °C until use. Aliquots were thawed once immediately before use and were not refrozen.

### 4.3. Nanoparticle Tracking Analysis

Isolated sEVs were diluted in 1 mL of distilled water, and particle size distribution was measured using a NanoSight NS300 system (Malvern, Malvern, UK). Data were analyzed using NTA software version 3.2.

### 4.4. Cryo-Electron Microscopy

For cryo-EM, 4 μL of each sample was applied to glow-discharged Quantifoil R 1.2/1.3 300-mesh holey carbon grids (Quantifoil, Großlöbichau, Germany) using a Vitrobot Mark IV (Thermo Fisher Scientific) under 100% humidity at 4 °C with a blotting time of 4 s. The prepared grids were transferred into an Elsa Cryo-Transfer Holder 698 (Gatan, Pleasanton, CA, USA) and imaged at 100 kV using a Tecnai 10 TEM (FEI, Hillsboro, OR, USA). Images were recorded using an UltraScan CCD camera (Gatan) at a nominal magnification of 40,000×. All TEM and cryo-EM analyses were performed at the Kangwon Center for Systems Imaging (Chuncheon, Republic of Korea) [55].

### 4.5. DiI Staining

To label sEVs, 1,1′-dioctadecyl-3,3,3′,3′-tetramethylindocarbocyanine perchlorate (DiI) was added to serum-free medium at a final concentration of 100 nM/mL. When cells reached approximately 80% confluence, the culture medium was replaced with DiI-containing medium, and the cells were incubated for 24 h.

After incubation, conditioned medium was collected and centrifuged at 1000× *g* for 10 min at 4 °C to remove cell debris. The supernatant was centrifuged at 10,000× *g* for 35 min at 4 °C to remove large vesicles and was then concentrated using a 100 kDa Amicon filter (Merck). The filtered medium was ultracentrifuged at 100,000× *g* for 70 min at 4 °C, and the sEV pellet was resuspended. Purified DiI-labeled sEVs were used to treat cells, which were observed using fluorescence microscopy.

### 4.6. Differentiation Assay

To investigate the effects of cancer cell-derived sEVs on C2C12 myoblast differentiation, cells were seeded at 1 × 10^5^ cells/well in 12-well plates and incubated for 24 h. The culture medium was then replaced with DM containing sEVs from MC38, CT26, MCA205, or B16F10 cells at a concentration of 20 μg/mL, corresponding to 1.00 × 10^10^, 3.05 × 10^9^, 1.91 × 10^9^, and 2.18 × 10^9^ particles/mL, respectively. The medium was changed every two days, and differentiation was assessed on days 2, 4, and 6 by immunofluorescence staining. For each time point, cells were cultured in separate wells and independently harvested for analysis, such that measurements at days 2, 4, and 6 were obtained from distinct wells rather than by repeated sampling of the same wells. Myotube area and diameter were quantified, and the expression levels of MRFs and MHC isoforms were examined by qRT-PCR.

### 4.7. Immunofluorescence Staining

To analyze C2C12 myotube formation, cells were fixed with 4% paraformaldehyde for 30 min at room temperature and washed three times with PBS. Fixed cells were permeabilized with 0.5% Triton X-100 in PBS on ice for 15 min and then washed five times with PBS. Blocking was performed using 1% bovine serum albumin in PBS for 1 h at room temperature to minimize nonspecific binding.

For immunostaining, cells were incubated overnight at 4 °C with an anti-MHC antibody (MF-20; Developmental Studies Hybridoma Bank, Iowa City, IA, USA) diluted 1:100 in 1% BSA/PBS. After three washes with PBS, cells were incubated for 1 h at room temperature with Cy3-AffiniPure Rabbit Anti-Mouse IgG (H + L) (Jackson ImmunoResearch, West Grove, PA, USA) diluted 1:500 in 1% BSA/PBS. Nuclear staining was performed using NucBlue Fixed Cell ReadyProbes Reagent (DAPI; Thermo Fisher Scientific) according to the manufacturer’s protocol. Stained cells were washed three times with PBS, dried, and imaged using a BX53 fluorescence microscope (Olympus, Tokyo, Japan). Myotube diameter and area were quantified using Cytovision software Version 7.3.1 Build 16 (Leica Biosystems, Deer Park, IL, USA). Cells were cultured in separate wells for each differentiation time point (days 2, 4, and 6). Three images were analyzed per biological replicate, and measurements were averaged within each biological replicate for statistical analysis.

### 4.8. Total RNA Isolation and qRT-PCR

Total RNA was extracted from sEV-treated differentiated C2C12 myotubes using PURY RNA Plus (GenDEPOT, Katy, TX, USA) according to the manufacturer’s protocol. RNA concentration was measured using a Nanodrop spectrophotometer (OPTIZEN nanoQ; KLAB, Daejeon, Republic of Korea), and 250 ng of total RNA was reverse-transcribed using HyperScript 2X RT Master Mix (GeneAll, Seoul, Republic of Korea). qRT-PCR was performed using the SensiFAST SYBR Lo-ROX Kit (Bioline, London, UK) on a QuantStudio 3 Real-Time PCR Instrument (Thermo Fisher Scientific). Three independent biological replicates were analyzed, with four technical replicate wells per biological replicate; technical replicates were averaged for statistical analysis. The PCR conditions were as follows: initial denaturation at 95 °C for 2 min, followed by 40 cycles of 95 °C for 5 s, primer-specific annealing at the temperatures listed in Table 2 for 10 s, and 72 °C for 10 s. Melt-curve analysis was performed to confirm amplification specificity. Primer sequences are listed in Table 2. GAPDH was used as the internal reference gene, and relative mRNA expression levels were calculated using the 2^−ΔΔCt^ method.

### 4.9. Western Blotting

For sEV analysis, 50 μg of total protein was mixed with sample buffer and denatured at 100 °C for 15 min. Samples were separated on 10% SDS-polyacrylamide gels and transferred to PVDF membranes (0.45 μm; Immobilon-P; Thermo Fisher Scientific). Membranes were blocked with 5% skim milk in Tris-buffered saline containing 0.2% Tween-20 (TBST) for 1 h at room temperature. Primary antibodies against Alix (Cell Signaling Technology, Danvers, MA, USA, #2171, RRID: AB_2299455), TSG101 (Santa Cruz Biotechnology, Dallas, TX, USA, sc-7964, RRID: AB_671392), Calnexin (Cell Signaling Technology, #2679, RRID: AB_2228381), CD63 (Santa Cruz Biotechnology, sc-5275, RRID: AB_627877), and CD9 (Santa Cruz Biotechnology, sc-13118) were diluted 1:1000 in TBST containing 1% skim milk and incubated overnight at 4 °C. After three washes with TBST, membranes were incubated with a goat anti-mouse IgG (H + L) HRP-conjugated secondary antibody (Cat. No. 31430; Invitrogen, Waltham, MA, USA, RRID: AB_228307) diluted 1:10,000 in 1% skim milk for 1 h at room temperature. Following three additional washes, protein bands were detected using ECL solution (Absignal, Seoul, Republic or Korea) and visualized using a WSE-6200 LuminoGraph II (ATTO, Tokyo, Japan). Ponceau S staining was used to assess total protein loading.

### 4.10. RP-Nano LC-ESI-MS/MS Analysis

A Thermo Scientific Quadrupole-Orbitrap instrument equipped with a Dionex U 3000 RSLCnano HPLC system was used. Mass spectrometric analyses were performed using an Orbitrap Exploris 240 mass spectrometer (Thermo Scientific).

Fractions were reconstituted in solvent A (water/acetonitrile, 98:2, *v*/*v*, containing 0.1% formic acid) and injected into the LC-nano ESI-MS/MS system. Samples were first trapped on an Acclaim PepMap 100 trap column (100 μm × 2 cm, nanoViper C18, 5 μm, 100 Å; Thermo Scientific, part number 164564) and washed for 6 min with solvent A at a flow rate of 4 μL/min. Samples were then separated on a PepMap RSLC C18 column (75 μm × 15 cm, nanoViper C18, 3 μm, 100 Å; Thermo Scientific, part number ES900) at a flow rate of 300 nL/min. The LC gradient was run from 2% to 8% solvent B over 10 min, from 8% to 30% over 55 min, followed by 90% solvent B (100% acetonitrile containing 0.1% formic acid) for 4 min, and finally 2% solvent B for 20 min.

Xcalibur software version 4.4 was used to collect MS data. The Orbitrap analyzer scanned precursor ions over a mass range of 350–1800 m/z at a resolution of 60,000 at *m*/*z* 200. Mass data were acquired automatically using Proteome Discoverer 2.5 (Thermo Scientific).

### 4.11. Statistical Analysis

All statistical analyses were performed using GraphPad Prism 5.0 (GraphPad Software, San Diego, CA, USA). Data are presented as the mean ± standard error of the mean (SEM). All experiments were independently performed three times (*n* = 3 independent experiments), unless otherwise indicated in the corresponding figure legends. One-way analysis of variance (ANOVA) was used to compare multiple groups, followed by Dunnett’s multiple-comparison post hoc test. Statistical significance was set at *p* < 0.05. For functional enrichment analysis, the Database for Annotation, Visualization, and Integrated Discovery (DAVID) was used to classify shared sEV proteins according to biological process, cellular component, molecular function, and KEGG pathway categories. Enriched terms with *p* < 0.001 were considered statistically significant.

## 5. Conclusions

In this exploratory in vitro study, treatment with cancer cell-derived sEVs was associated with reduced C2C12 myotube formation and altered expression patterns of MRFs and MHC isoforms in a time- and sEV origin-dependent manner. These findings suggest that cancer cell-derived sEVs may contribute to impaired myoblast differentiation and provide evidence for a potential role of cancer-derived extracellular vesicles in the regulation of skeletal muscle differentiation. Further studies using independent sEV preparations, appropriate biological controls, protein-level validation, functional differentiation endpoints, and in vivo models will help elucidate the underlying mechanisms and determine the physiological relevance of these findings.

## Figures and Tables

**Table 1:** Gene IDs related to the proteasome pathway identified among shared sEV proteins.

Entrez Gene ID	Gene Name	Species
19179	protease (prosome, macropain) 26S subunit, ATPase 1 (*Psmc1*)	*Mus musculus*
19181	proteasome (prosome, macropain) 26S subunit, ATPase 2 (*Psmc2*)	*Mus musculus*
19182	proteasome (prosome, macropain) 26S subunit, ATPase 3 (*Psmc3*)	*Mus musculus*
23996	proteasome (prosome, macropain) 26S subunit, ATPase, 4 (*Psmc4*)	*Mus musculus*
67089	proteasome (prosome, macropain) 26S subunit, ATPase, 6 (*Psmc6*)	*Mus musculus*
70247	proteasome (prosome, macropain) 26S subunit, non-ATPase, 1 (*Psmd1*)	*Mus musculus*
69077	proteasome (prosome, macropain) 26S subunit, non-ATPase, 11 (*Psmd11*)	*Mus musculus*
59029	proteasome (prosome, macropain) 26S subunit, non-ATPase, 14 (*Psmd14*)	*Mus musculus*
22123	proteasome (prosome, macropain) 26S subunit, non-ATPase, 3 (*Psmd3*)	*Mus musculus*
66413	proteasome (prosome, macropain) 26S subunit, non-ATPase, 6 (*Psmd6*)	*Mus musculus*
17463	proteasome (prosome, macropain) 26S subunit, non-ATPase, 7 (*Psmd7*)	*Mus musculus*
57296	proteasome (prosome, macropain) 26S subunit, non-ATPase, 8 (*Psmd8*)	*Mus musculus*
19170	proteasome (prosome, macropain) subunit, beta type 1 (*Psmb1*)	*Mus musculus*
26445	proteasome (prosome, macropain) subunit, beta type 2 (*Psmb2*)	*Mus musculus*
19172	proteasome (prosome, macropain) subunit, beta type 4 (*Psmb4*)	*Mus musculus*

**Table 2:** Primer sequences used for mouse MHC and MRF genes (5′ → 3′).

Gene	Gene	Sequence (5′ → 3′)	Tm	Reference
*Pax7*	Forward	ACC AGT ACA GCC AGT ATG GCC A	60 °C	[56]
*Pax7*	Reverse	GTG TTC CCC AAG CTT CAT ACG G	60 °C	
*MyoG*	Forward	CCC ATG GTG CCC AGT GAA	57.2 °C	
*MyoG*	Reverse	GCA GAT TGT GGG CGT CTG TA	57.2 °C	
*MyoD*	Forward	GGC AGA ATG GCT ACG ACA CCG	60 °C	
*MyoD*	Reverse	CTT CCC TGG CCT GGA CTC GC	60 °C	
*myf5*	Forward	GAA CAG CAG CTT TGA CAG CAT	60 °C	
*myf5*	Reverse	AAT GCT GGA CAA GCA TCC AA	60 °C	
*Mrf4*	Forward	CTA CAT TGA GCG TCT ACA GGA CC	54.8 °C	[57]
*Mrf4*	Reverse	CTG AAG ACT GCT GGA GGC TG	54.8 °C	
*MHC* *I*	Forward	CTC AAG CTG CTC AGC AAT CTA TTT	56.5 °C	[58]
*MHC* *I*	Reverse	GGA GCG CAA GTT TGT CAT AAG T	56.5 °C	
*MHC* *II* *a*	Forward	AGG CGG CTG AGG AGC ACG TA	61.5 °C	
*MHC* *II* *a*	Reverse	GCG GCA CAA GCA GCG TTG G	61.5 °C	
*MHC* *II* *b*	Forward	CAA TCA GGA ACC TTC GGA ACA C	59 °C	
*MHC* *II* *b*	Reverse	GTC CTG GCC TCT GAG AGC AT	59 °C	
*MHC* *II* *x*	Forward	GAG GGA CAG TTC ATC GAT AGC AA	58.3 °C	
*MHC* *II* *x*	Reverse	GGG CCA ACT TGT CAT CTC TCA T	58.3 °C	
*GAPDH*	Forward	AGG TCG GTG TGA ACG GAT TTG	57.2 °C	[59]
*GAPDH*	Reverse	TGT AGA CCA TGT AGT TGA GGT CA	57.2 °C	

## Data Availability

The original contributions presented in this study are included in the article/Supplementary Materials. The raw mass spectrometry data have been deposited in the PRIDE repository (Accession number: PXD082913). Further inquiries can be directed to the corresponding author.

## Supplementary Materials

The following supporting information can be downloaded at: https://www.mdpi.com/article/10.3390/ijms27177894/s1.
