# Supplementary material for: Cancer Cell-Derived Small Extracellular Vesicles Are Associated with Impaired C2C12 Myoblast Differentiation
Source: Int J Mol Sci. 2026 Sep 4;27(17):7894. doi: 10.3390/ijms27177894 (PMC13566097; doi:10.3390/ijms27177894)
Supplement: Supplementary file 1 [file ijms-27-07894-s001.zip › ijms-4414834-supplementary.pdf]

sEV 50ug

Ponceau S staining

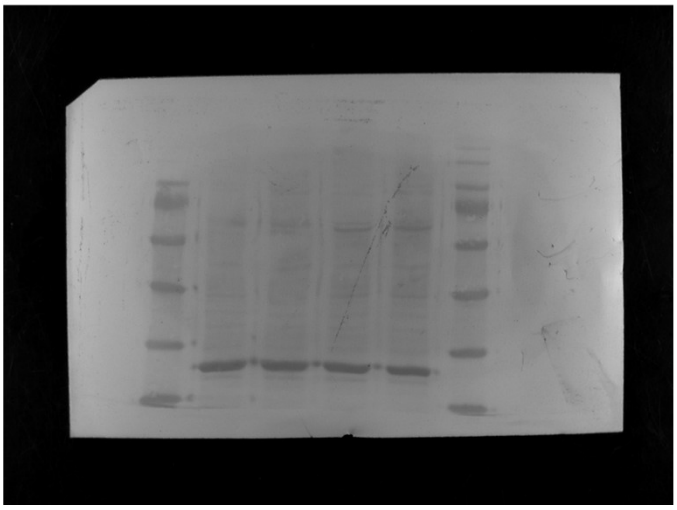

Supplementary Figure S1. Ponceau S staining of sEV samples. Ponceau S staining was performed to assess total protein loading of sEV samples used for downstream analyses.

A

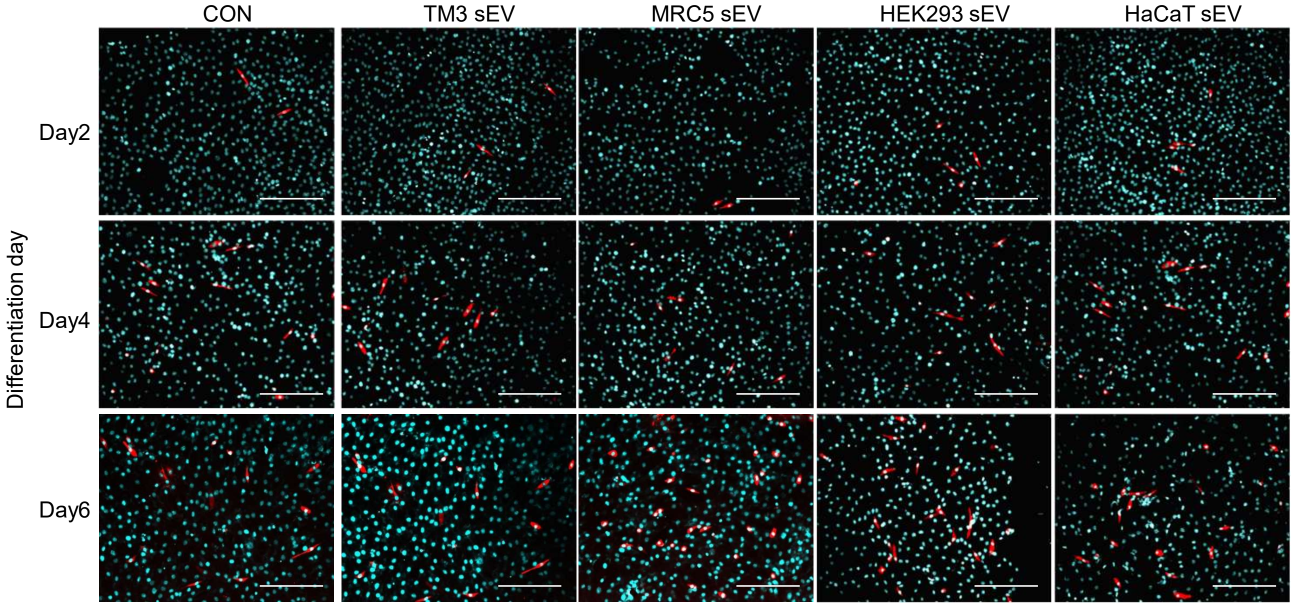

B

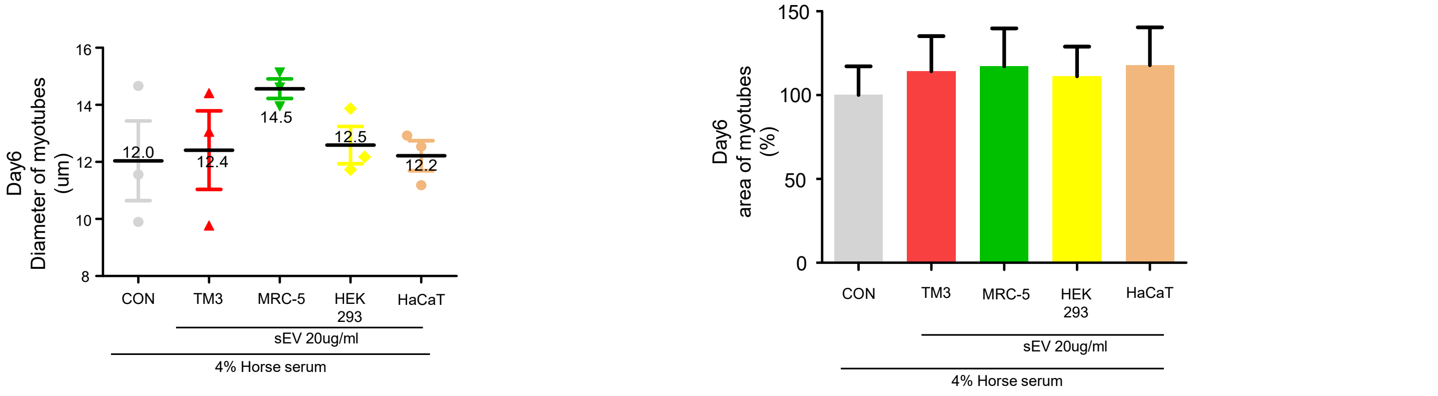

Supplementary Figure S2. Effects of non-cancer cell-derived sEVs on myogenic differentiation of C2C12 myoblasts
(A) Representative immunofluorescence images of C2C12 myoblasts treated with non-cancer cell-derived sEVs and stained for myosin heavy chain (MHC). Nuclei were counterstained with DAPI. Scale bar, 200 µm. (B) Quantification of MHC-positive myotube formation. (C) Quantification of myotube area. Data are presented as mean ± SD from three independent biological replicates (*n* = 3), with three images analyzed per biological replicate (nine images in total). Statistical significance was determined using [statistical test]. $p < 0.05$, $p < 0.01$, $p < 0.001$

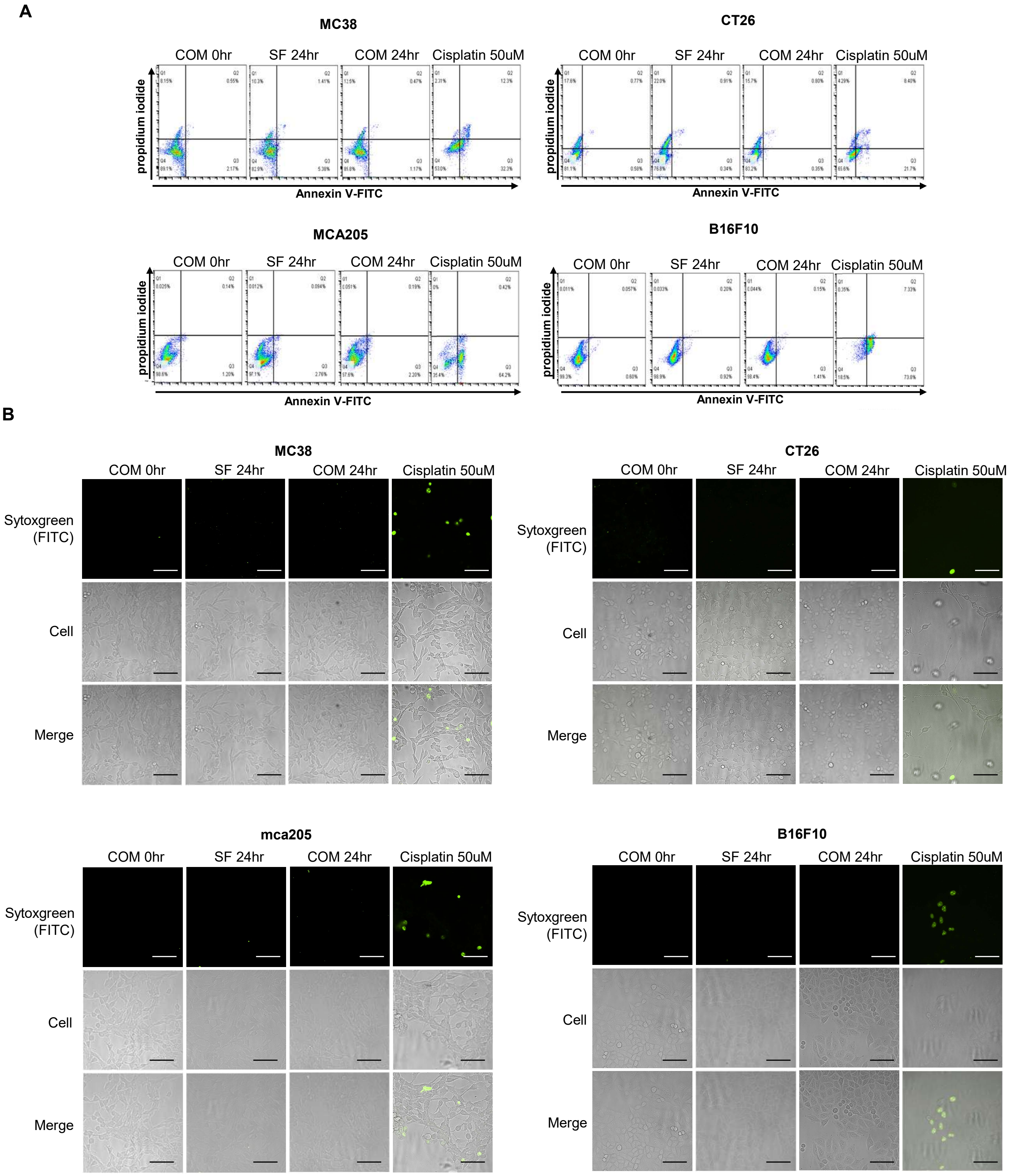

Supplementary Figure S3. Assessment of apoptosis and cell death under serum-free culture conditions. (A) Annexin V-FITC staining was performed to assess apoptosis under serum-free culture conditions. Cells maintained in complete medium for 24 h (COM 24 h) or cultured in serum-free medium for 24 h (SF 24 h) were compared, with cisplatin-treated cells included as a positive control for apoptosis. (B) SYTOX Green staining was performed to assess cell death under serum-free culture conditions. Cells were cultured in complete medium (COM) or serum-free medium (SF) for 24 h, with cisplatin (50 μM) used as a positive control. SYTOX Green was diluted 1:20,000, and fluorescence images were acquired in the FITC channel. Scale bar, 100 μm.
